# The Effects of Chemotherapy-Induced Polyneuropathy on Postural Balance

**DOI:** 10.7759/cureus.16617

**Published:** 2021-07-25

**Authors:** Mariah Gleeson, Lori Boright, Joshua Haworth

**Affiliations:** 1 Human Movement Science, Oakland University, Rochester, USA; 2 Physical Therapy, Oakland University, Rochester, USA

**Keywords:** falls, polyneuropathy, postural balance, btracks, tug

## Abstract

While cancer prevalence and survival rates are increasing, cancer-treatment-related functional decline and decreased quality of life are on the rise. Adverse side effects such as polyneuropathy are commonly associated with neurotoxic chemotherapeutic agents (i.e., taxanes or platinums) resulting in a decrease in postural balance and an increased risk for falls. This case report highlights an adult with polyneuropathy who received five months of chemotherapy treatment for a breast cancer diagnosis. Measurements included sensory integration of balance control using the BTrackS modified Clinical Test of Sensory Integration and Balance (mCTSIB) protocol and the Timed Up and Go (TUG) test. Compared to normative data, the participant demonstrated poor balance control in all conditions. Specifically, the results indicated proprioception was the most compromised component of balance control. The participant also demonstrated a slower TUG time compared to women of similar age. Our findings highlight the value of quantitative assessment of balance control following chemotherapy. More research would be needed to further support the tailored therapeutic approaches to prevent falls following chemotherapy treatment.

## Introduction

Advancements in early cancer detection and treatments have precipitated an increase in cancer prevalence and five-year survival rate. In 2019, 16.9 million individuals in the United States were living with a history of cancer. Furthermore, 3.8 million women were living with a history of invasive breast cancer in 2019 [1]. A common treatment for breast cancer is neurotoxic chemotherapy. Agents such as taxanes and platinums are commonly associated with chemotherapy-induced peripheral neuropathy (CIPN). While CIPN refers to damage of one peripheral nerve, polyneuropathy may better represent the symptom. Polyneuropathy is damage to the peripheral nervous system (PNS) consisting of sensory and/or motor symptoms of varying severities, negatively affecting one’s quality of life (QOL) [2].

Sensory symptoms of polyneuropathy may include a decline in proprioception resulting from numbness of the distal extremities with tingling or stinging, whereas motor symptoms may include muscular weakness. Collectively, sensory and motor symptoms may decrease postural balance and increase falls risk [3]. The severity of polyneuropathy is dependent on treatment type and cumulative dose of neurotoxic chemotherapy. Reciprocally, polyneuropathy may affect treatment outcomes due to dose compromise (e.g., delays, reduction, or discontinuation) [2]. Clinical risk factors placing individuals at higher risk of developing polyneuropathy include neuropathy at baseline, older age, diabetes, and smoking. Polyneuropathy may last weeks to months after the first treatment. After completion of treatment 30% of individuals still experience symptoms at six months or beyond [4].

About one in four older adults (65 years or older) experience a fall each year [5]; moreover, women with chemotherapy-induced polyneuropathy symptoms may experience a 24% increase in falls compared to older adults [6]. Common risk factors associated with falls are age-related physiological changes [7], post-treatment [8], and chemotherapy-induced polyneuropathy [8]. The relation between chemotherapy-induced polyneuropathy and falls has been well confirmed among clinical settings [6,8,9]. From a clinical perspective, it is essential to establish a threshold for early identification in declines in balance and implement balance and strength training programs for individuals more susceptible to functional decline. There is paucity in the literature regarding the use of the BTrackS modified Clinical Test of Sensory Integration and Balance (mCTSIB) in chemotherapy-induced polyneuropathy. The mCTSIB is used to specifically probe the role of vision, proprioception, and vestibular sensitivities in the control of balance. Therefore, the purpose of this case report is to measure static and dynamic balance using BTrackS mCTSIB and the Timed Up and Go (TUG) in an adult with polyneuropathy.

## Case presentation

This case report highlights a 57-year-old woman who received five months of chemotherapy for breast cancer completed in 2016. The participant’s Functional Assessment of Cancer Therapy/Gynecologic Oncology Group - Neurotoxicity (FACT/GOG-Ntx) score was 133/152. She specifically reported tightness and discomfort in her hands, lower extremity joint pain, muscle cramps, and generalized weakness. She reported no comorbidities associated with polyneuropathy.

Force plates are the gold standard measurement for assessing postural balance [10]. The BTrackS is a low-cost, portable force plate that is accurate and reliable. The BTrackS mCTSIB protocol quantifies balance by tracking the center of pressure (CoP) during various sensory manipulating conditions. The measured path length represents the total horizontal distance (front to back and side to side) travelled by the CoP. Large values of path length indicate more movements, suggesting less control of the maintenance of balance. The mCTSIB protocol measures standard, proprioception, vision, and vestibular contributions toward balance [11]. The protocol consists of four 20-second trials with breaks between each trial (< 10s), standing as still as possible with their hands on their hips and feet shoulder-width apart. In the first trial, the participant stood with their eyes open measuring the ‘standard’ condition, or uncompromised condition. In the second trial, the participant stood with their eyes closed measuring the ‘proprioception’ condition. For the third trial, the participant stood on a foam pad with their eyes open measuring the ‘vision’ condition. In the fourth and final trial, the participant stood on a foam pad with their eyes closed measuring the ‘vestibular’ condition. To better understand the balance measures of the case participant, we compared the results to normative data of a healthy sample of similarly aged women [11].

The TUG is a reliable and valid measurement for dynamic balance and may be useful to identify clinical changes over time [12]. The participant was instructed to start seated in a standard height chair, stand up, walk 3m, turn around, and sit back down. Time started when the researcher said “go,” and ended when the participant’s glutes touched the seat of the chair. Time was measured using a stopwatch to the nearest 0.1s [12].

Outcomes 

**Figure 1:**
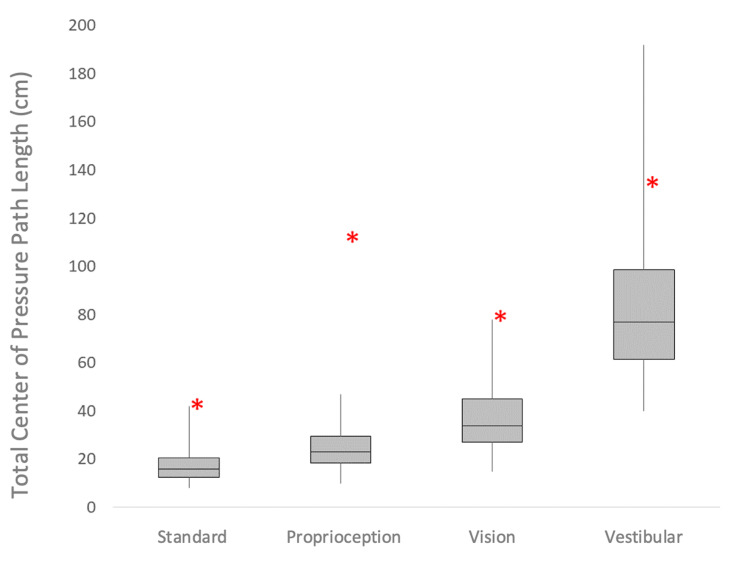
Case Report Compared to Normative Data Case participant data (asterisk) against normative data (box plot) adapted from Goble et al. [11]. The distal ends of the whiskers represent the 1st and 99th percentile values from the normative data reported. The 1st and 3rd quartiles were calculated, and the 2nd quartile extracted directly from the data report.

The participant demonstrated poor balance control in all conditions. She had a path length (cm) in standard (eyes open/firm) of 43, proprioception (eyes closed/firm) 110, vision (eyes open/foam) 81, and vestibular (eyes closed/foam) 133. In comparison to normative data for 50- to 59-year-old women (Figure 1) [11], the participant’s path length was between the 1st and 10th percentile in the vestibular condition, and below the 1st percentile in standard and vision conditions. Moreover, the results indicate the proprioception contribution toward balance control was severely compromised, with the participant’s path length in this condition more than double the largest values of the normal range. The participant also had an 8.28s TUG time.

## Discussion

It has been confirmed that individuals with chemotherapy-induced polyneuropathy symptoms present a decrease in postural balance and an increase in fall risk [6,8,9]. Various underlying physiological mechanisms may explain a compromised proprioception system in chemotherapy-induced polyneuropathy. Most chemotherapy agents directly or indirectly affect the dorsal root ganglia (DRG), resulting in peripheral nerve vulnerability [13]. The DRG sends information from the PNS to the central nervous system (CNS) and is associated with neuropathic pain [14]. Neurotoxic chemotherapeutic agents associated with polyneuropathy (e.g., taxanes and platinums) and their various underlying physiological mechanisms are not fully elucidated [13], however changes such as oxidative stress, axon degeneration, and inflammation are associated with neural dysfunction [15]. It is perhaps for this reason, there is a decrease in proprioception and postural balance. 

For the BTrackS measures, our results showed the participant is between the 1st and 10th percentile in vestibular system (eyes closed/foam), and the proprioception (eyes closed/firm) system is severely compromised. Our findings are consistent with prior studies showing balance deficits and postural instability when manipulating proprioception [16,17]. Our participant demonstrated a slower TUG time than expected in comparison to Isles et al. TUG (6.44 ±0.17s) normative values in 50- to 59-year-old community-dwelling women [18]. The reason for the discrepancy in TUG times may be due to variations in clinical characteristics. Overall, the combination of the FACT/GOG-Ntx and TUG scores indicate that she does not perceive a mobility and QOL burden, however objectively it must be noted that TUG score falls out of range when age-matched to her peers indicating a potential higher fall risk. Limitations of this study are that the specific chemotherapy treatment regimen was unknown, and the case report does not generalize all individuals with polyneuropathy. The strengths of this study include the use of a gold standard measurement - force plate, and the previous work done with BTrackS to establish the normative data provided by Goble et al. [11]. 

## Conclusions

From a clinical perspective, it is important to establish early balance assessments to detect abnormalities and/or decline in persons diagnosed with cancer to decrease the risk for falls. Doing so will improve patient care and guide clinicians to implement appropriate balance and strength training programs. The BTrackS mCTSIB and TUG indicate not only high risk for falls, but the identification of a decrease in specific sensory conditions that could be used to define therapeutic approach. Thus, it seems that mCTSIB would be an important clinical measurement in the early assessment of balance. For future research, studies with a larger sample size, and interventional studies to elucidate postural instability with polyneuropathy are supported by our findings. This study supports the feasibility of using BTrackS to assess the sensory contributions to balance of persons who are post-treatment and may be an important clinical assessment due to proprioception being extremely compromised.
